# Determinants of outpatient expenditure within primary care in the Brazilian National Health System: Determinantes de gastos ambulatoriais na atenção primária do sistema público de saúde brasileiro

**DOI:** 10.1590/1516-3180.2016.0224141116

**Published:** 2017-04-03

**Authors:** Bruna Camilo Turi, Jamile Sanches Codogno, Flávia Mori Sarti, Nana Kwame Anokye, Rômulo Araújo Fernandes, Henrique Luiz Monteiro

**Affiliations:** I MSc, PhD. Researcher, Postgraduate Program on Kinesiology, Universidade Estadual Paulista (UNESP), Rio Claro (SP), Brazil.; II MSc, PhD. Professor, Department of Physical Education, Universidade Estadual Paulista (UNESP), Presidente Prudente (SP), Brazil.; III MSc, PhD. Professor, Department of Food and Experimental Nutrition, Universidade de São Paulo (USP), São Paulo (SP), Brazil.; IV PhD. Senior Research Fellow. Institute of Environment, Health and Societies, Brunel University, Uxbridge, London, United Kingdom.; V MSc, PhD. Professor, Department of Physical Education, Universidade Estadual Paulista (UNESP), Bauru (SP), Brazil.

**Keywords:** Health expenditures, Primary health care, Public health, Risk factors, Epidemiology

## Abstract

**CONTEXT AND OBJECTIVE:**

One of the big challenges facing governments worldwide is the financing of healthcare systems. Thus, it is necessary to understand the factors and key components associated with healthcare expenditure. The aim here was to identify demographic, socioeconomic, lifestyle and clinical factors associated with direct healthcare expenditure within primary care, among adults attended through the Brazilian National Health System in the city of Bauru.

**DESIGN AND SETTING:**

Cross-sectional study conducted in five primary care units in Bauru (SP), Brazil.

**METHODS:**

Healthcare expenditure over the last 12 months was assessed through medical records of adults aged 50 years or more. Annual healthcare expenditure was assessed in terms of medication, laboratory tests, medical consultations and the total. Body mass index, waist circumference, hypertension, age, sex, physical activity and smoking were assessed through face-to-face interviews.

**RESULTS:**

The total healthcare expenditure for 963 participants of this survey was US$ 112,849.74 (46.9% consultations, 35.2% medication and 17.9% laboratory tests). Expenditure on medication was associated with overweight (odds ratio, OR = 1.80; 95% confidence interval, CI: 1.07-3.01), hypertension (OR = 3.04; 95% CI: 1.91-4.82) and moderate physical activity (OR = 0.56; 95% CI: 0.38-0.81). Expenditure on consultations was associated with hypertension (OR = 1.67; 95% CI: 1.12-2.47) and female sex (OR = 1.70; 95% CI: 1.14-2.55).

**CONCLUSIONS:**

Our results showed that overweight, lower levels of physical activity and hypertension were independent risk factors associated with higher healthcare expenditure within primary care.

## RESUMO

**CONTEXTO E OBJETIVO:**

Um dos grandes desafios dos governos em todo o mundo é o financiamento de sistemas de saúde e, por isso, é necessário compreender fatores e componentes-chave associados a despesas em saúde. O objetivo foi identificar fatores demográficos, socioeconômicos, de estilo de vida e clínicos associados aos gastos diretos com saúde na atenção primária entre adultos do Sistema Único de Saúde da cidade de Bauru.

**TIPO DE ESTUDO E LOCAL:**

Estudo transversal realizado em cinco Unidades Básicas de Saúde em Bauru (SP), Brasil.

**MÉTODO:**

Gastos com saúde nos últimos 12 meses foram avaliados através de prontuários médicos de adultos de 50 anos ou mais. Gastos anuais com saúde foram avaliados com: medicamentos, exames laboratoriais, consultas médicas e total. Índice de massa corporal, circunferência da cintura, hipertensão, idade, sexo, atividade física e tabagismo foram avaliados por meio de entrevista face a face.

**RESULTADOS:**

O gasto total com serviços de saúde para os 963 participantes deste inquérito foi de US$ 112.849.74 (46,9% consultas, 35,2% medicamentos e 17,9% exames). Gastos com medicamentos foram associados com sobrepeso (*odds ratio*, OR = 1,80 [intervalo de confiança, IC 95%: 1,07-3,01]), hipertensão (OR = 3,04 [IC 95%: 1,91-4,82]) e atividade física moderada (OR = 0,56 [95% IC: 0,38-0,81]). Gastos com consultas foram associados com hipertensão (OR = 1,67 [IC 95%: 1,12-2,47]) e sexo feminino (OR = 1,70 [IC 95%: 1,14-2,55]).

**CONCLUSÃO:**

Nossos resultados mostraram que sobrepeso, menor nível de atividade física e hipertensão são fatores de risco independentes associados com maiores gastos com saúde na atenção primária.

## INTRODUCTION

One of the big challenges facing governments worldwide is the financing of healthcare systems. The challenge is more difficult in developing nations, not only due to limited budgets, but also because of the increasing prevalence of chronic diseases.^1^^,^^2^^,^^3^ In 2005, cardiovascular diseases and diabetes mellitus were the leading causes of mortality in developing nations, accounting for 30% of deaths.^1^ However, in 2012, among the 56 million deaths that occurred, 67.8% were due to chronic diseases, especially cardiovascular and respiratory diseases and cancer.^4^

In Brazil, treatment of chronic cases relating to overweight/obesity in the adult population alone cost US$ 2.1 billion in 2008, i.e. around 1% of gross domestic product (GDP).^2^ The cumulative losses of GDP from 2006 to 2015 due to all chronic diseases were US$ 4.2 billion.^1^ In terms of economic impact, the burden of chronic diseases on the Brazilian economy is significant because the Brazilian National Health System grants free access to healthcare services at all levels of complexity to the entire population. In 2011, the costs relating to maintenance of the Brazilian National Health System accounted for 9% of the national GDP.^5^

The growing public healthcare expenditure in Brazil has captured the attention of the Brazilian government. The need for development of preventive strategies to address this issue has been highlighted. However, as far as we know, there is a lack of evidence informing the progress of such policies. Previous studies have examined the association between higher healthcare expenditure and specific determinants, such as physical inactivity, obesity and arterial hypertension.^2^^,^^6^^,^^7^ However, the effects of these variables on healthcare expenditure are usually assessed separately and it is not clear which one is the most relevant as a cause of the rising healthcare expenditure in Brazil. Therefore, identification of correlates of higher healthcare expenditure among the adult population is an important step towards elaborating effective strategies for braking this increasing trend.

To formulate effective policies for addressing the economic burden, not only the drivers of rising costs but also the key components associated with healthcare expenditure need to be understood. These include medication dispensed, laboratory tests and consultations, given that the dynamics of costs may vary according to the type of service.^8^ Moreover, even though Brazil is a nation of continental proportions, with huge metropolises like São Paulo and Rio de Janeiro, the largest proportion of the Brazilian population lives in small and medium-sized cities. Recent surveys have shown that these adults living in medium-sized Brazilian cities are widely affected by chronic diseases and other health problems.^9^^,^^10^^,^^11^ Therefore, in terms of the burden on healthcare costs, small and medium-sized cities are important within the dynamics of healthcare costs in Brazil. However, data on healthcare costs and their correlates in this setting are scarce.

## OBJECTIVE

The objective of this study was to identify demographic, socioeconomic, lifestyle and clinical factors associated with direct healthcare expenditure within primary care among adults who were attended through the Brazilian National Health System and who were living in Bauru, a medium-sized city in the state of São Paulo.

## METHODS

### Study design and population

The data were collected through a cross-sectional study carried out in 2010 (August to December 2010) in the city of Bauru, which has around 360,000 inhabitants and is located in the central region of the state of São Paulo, Brazil. In this study, the minimum sample size required was 882 individuals. The sample size was estimated based on the percentage of adults attended through the Brazilian National Health System who were classified as “high cost” (25%), error of 3.5%, alpha error of 5% (Z = 1.96) and design effect of 50%. The percentage of 25% was arbitrary and came from previous papers,^6^^,^^7^ in which it had been used because of the absence of previous data about this issue.

Details about the sampling process can be found in previous papers.^6^^,^^7^^,^^12^ Briefly, the Municipal Health Department was contacted, the researchers presented the objectives of the research project and asked for permission to access the patients’ medical records. After the Research Ethics Board of São Paulo State University, Bauru campus, had assessed the project and approved it (procedural number 1046/46/01/10), the Health Department authorized access to all medical records within the primary healthcare services of the Brazilian National Health System.

The Brazilian National Health System is divided into primary, secondary and tertiary services. Primary services include preventive actions (e.g. vaccination) and treatment of chronic conditions (e.g. medication for patients with chronic diseases) and clinical consultations. Such services are provided at primary care facilities called primary healthcare units (PHUs), which provide different types of healthcare specialists (e.g. general practitioners, nurses and dentists) and have the capacity to implement low-complexity healthcare procedures for the community. In the city of Bauru, there are 17 PHUs, spread out across all geographical regions of the metropolitan area. In this survey, we only included traditional PHUs (without inclusion in the Family Health Strategy). The survey included five PHUs, i.e. one in each metropolitan area of Bauru (east, west, north, south and downtown). In selecting each PHU, the number of patients served was taken into consideration, i.e. the PHU with the highest number of patients registered was selected in each geographical region.

In each PHU, the participants were randomly selected using the identification number on the medical records. The SPSS software, version 13.0, was used for random sampling. Among the randomly selected medical records, the inclusion criteria were checked. Firstly, all subjects needed to be aged ≥ 50 years, because this age has been correlated with development of chronic diseases in the Brazilian population^8^^,^^11^ and is a cutoff point relating to significantly increased healthcare costs among adults.^13^ Secondly, all subjects needed to have attended at least one medical consultation during the last six months: this was used as an indicator of current residence in Bauru and usage of the healthcare system.

Patients who fulfilled both of these inclusion criteria were contacted by phone. Those who agreed to take part in the study were invited to come to the PHU for assessment. At the PHU, the participants signed a standard written consent form in which they agreed to participate in this study. They were then interviewed and anthropometric measurements were made.

## MEASUREMENTS

### Dependent variable

#### Annual healthcare expenditure

Using participants’ medical records (which were stored at the PHU), only the direct annual healthcare expenditure recorded over the last 12 months prior to the survey date were estimated. These estimates were based on the major components: medical consultations, medication dispensed and laboratory tests. At the PHU, in a reserved room, two trained researchers extracted data from the medical records. In the event of any doubts, the head nurse of the PHU was consulted. Public healthcare reimbursement values provided by the PHU office (administrated by the Municipal Finance Department) were used to calculate monetary values for the medication dispensed, laboratory tests done and consultations performed. For the tests and medical consultations, the exact value paid by the municipal administration was calculated using the receipts, while for medicine delivered to the patient, the amount was estimated, because the municipal administration pays for the entire pack of medicine, but in some cases releases less than the entire pack to the patient.

All expenditures were initially calculated in the Brazilian currency (real) and then converted to American dollars (US$) using the mean exchange rate for the period from January 2009 to December 2009. Finally, the monetary values were adjusted according to the annual inflation rate in Brazil observed over the period 2010-2015, so as to express the costs in a manner closer to current values. The inflation index used was IPCA-IBGE (Índice Nacional de Preços ao Consumidor Amplo, from Instituto Brasileiro de Geografia e Estatística).

Four dependent variables indicating different types of expenditure were specified:

1. medication for regular use (sporadic use of medications, such as anti-inflammatory drugs, was not assessed);
2. medical consultations;
3. laboratory tests; and
4. overall (sum of medications, consultations and laboratory tests).

The numbers of medications and consultations were registered and inserted in the multivariate models as a confounder.

### Independent variables

The same two trained researchers who were responsible for data collection from the medical records also conducted face-to-face interviews to assess the independent variables (including application of questionnaires and making anthropometric measurements). The independent variables were selected based on variables that had been shown to be correlated with healthcare expenditure in previous studies.^1^^,^^2^^,^^6^^,^^14^ Considering that this was the first study of its kind in Brazil, we also included other variables.

#### Anthropometric measurements and health status

Body mass index (BMI) was calculated using measurements of weight (digital scale with maximum capacity of 150 kilograms) and height (wall-mounted stadiometer with maximum height of 2 meters).^15^ Participants with BMI between 25 and 29.9 kg/m^2^ were considered overweight and obesity was defined as BMI ≥ 30 kg/m^2^.^16^ Waist circumference (WC) was used as a screening tool for abdominal obesity and the cutoff points were defined as 1.02 m for men and 0.88 m for women.^17^ Hypertension, diabetes mellitus and dyslipidemia were assessed as present based on diagnoses by physicians that were identified through the participants’ medical records.

#### Demographic and socioeconomic variables

Data on chronological age (categorized as < 65 years or ≥ 65 years because of its association with chronic diseases and higher healthcare costs)^6^^,^^7^^,^^11^ and sex were obtained from the medical records and verified through the interviews. Socioeconomic status was measured using a validated Brazilian family income questionnaire,^18^ which specifies the following income groups: low (classes C, D and E, i.e. family income of US$ 76.94-966.38 per month) and high (classes A and B, i.e. family income of US$ 1,823.33-2,703.61 per month). This questionnaire estimates income based on data on formal schooling, appliances available in the home and physical characteristics of the home (e.g. number of toilets).

#### Lifestyle behavior

Information regarding physical activity levels was collected using the questionnaire developed by Baecke et al.^19^ Individuals’ physical activity levels were expressed as the sum of scores for all specific domains of physical activity (i.e. occupational, active transportation and sport during leisure time). These scores were then divided into quartiles and the participants were classified into three groups: inactive (≤ P25), moderately active (< P25 and > P75) and sufficiently active (≥ P75).^6^^,^^7^^,^^14^ Smoking status was obtained through the interviews and was specified as “yes” (current smokers regardless of number of cigarettes per day) or “no” (former smokers or never smoked).

### Statistical analyses

Binary logistic regression was fitted to analyze the relationship between annual healthcare expenditure and health, demographic, socioeconomic and lifestyle indicators. In the binary logistic regression, all the models were adjusted using all the independent variables with statistical significance (P-value) < 0.05 from the chi-square test, plus the number of consultations, number of medicines and diagnoses of diabetes and dyslipidemia. The dependent variables were specified as binary variables, such that one indicated the highest quartile of expenditures (≥ P75), and zero indicated other levels. Regression models were fitted separately for each of the four dependent variables.

The magnitude of associations was assessed using odds ratios (OR) and their 95% confidence intervals (95% CI). The Hosmer-Lemeshow goodness-of-fit test was used to determine how well the model fitted the data (non-significant results indicated an adequate fit). Bivariate analyses were conducted to identify significant associations between dependent and independent variables. Categorical variables were expressed as rates and were compared using the chi-square test (Yates’s correction was applied in 2 x 2 contingency tables).

Due to nonparametric distributions, numerical variables were presented as medians and comparisons were made using the Kruskal-Wallis and Mann-Whitney tests. All statistical analyses were performed using BioEstat (release 5.0) and P-value significance was set at 0.05.

## RESULTS

The sample consisted of 963 participants, who were mainly women (73.4% versus 26.6%, P-value = 0.001), with ages ranging from 50 to 96 years. During the data collection, the loss rate was 49.7%, i.e. 1915 calls were made and 952 potential subjects were lost for different reasons (did not pick up the phone, incorrect number in the medical records, did not want to participate in the study, or scheduled for interview but did not show up).

The prevalence of hypertension was 76.8% (95% CI: 74.1 to 79.5), while diabetes mellitus and dyslipidemia were observed in 28.5% and 32.4% of the sample, respectively. We observed that 83.2% (CI: 80.8% to 85.5%) of the individuals had low income.

The total annual healthcare expenditure for the 963 participants of this survey was US$ 112,849.74 and the maximum amount spent on any patient was U$ 941.78 per year. Consultations with healthcare professionals accounted for the highest proportion of total public healthcare expenditure (46.9%; US$ 53,025.05), followed by medication dispensed (35.2%; US$ 39,774.90) and then laboratory tests (17.9%; US$ 20,040.79) (Table 1).

**Table 1:**
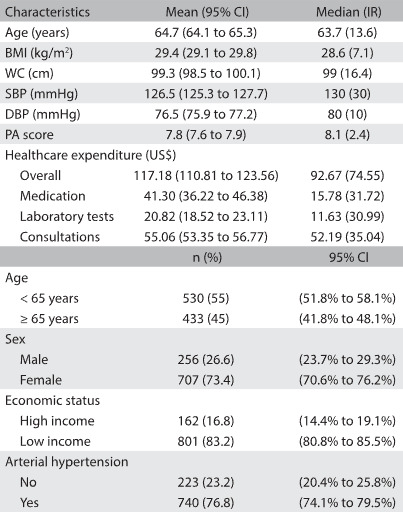
Characteristics of the sample in Bauru (SP), Brazil (n = 963)

BMI = body mass index; WC = waist circumference; SBP = systolic blood pressure; DBP = diastolic blood pressure; PA = physical activity; IR = interquartile range.

The different types of expenditure were found to be associated with different factors. Bivariate analysis showed that female participants were more likely to generate higher healthcare expenditure on laboratory tests (P-value = 0.022) and consultations (P-value = 0.005). Participants with hypertension had higher total healthcare expenditure (P-value = 0.001), especially on medication dispensed (P-value = 0.001) and consultations (P-value = 0.006) (Table 2). Smoking was associated with lower healthcare expenditures on consultations (P-value = 0.012). Presence of overweight or obesity was associated with higher healthcare expenditure in all the categories considered. Higher levels of physical activity were associated with lower healthcare expenditure on medication dispensed (P-value = 0.001) (Table 2).

**Table 2:**
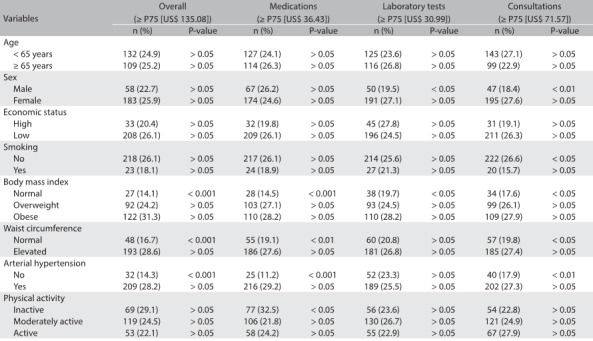
Variables associated with high public healthcare expenditure within primary care in Bauru (SP), Brazil (n = 963), from bivariate analysis

After accounting for potential confounders, expenditure on medication was found to be associated with overweight (OR = 1.80; 95% CI: 1.07 to 3.01), hypertension (OR = 3.04; 95% CI: 1.91 to 4.82) and moderate physical activity (OR = 0.56; 95% CI: 0.38 to 0.81). Expenditure on consultations was found to be associated with hypertension (OR = 1.67; 95% CI: 1.12 to 2.47). Overall healthcare expenditure was associated with female sex and hypertension (Table 3). All multivariate models were found to show adequate fit.

**Table 3:**
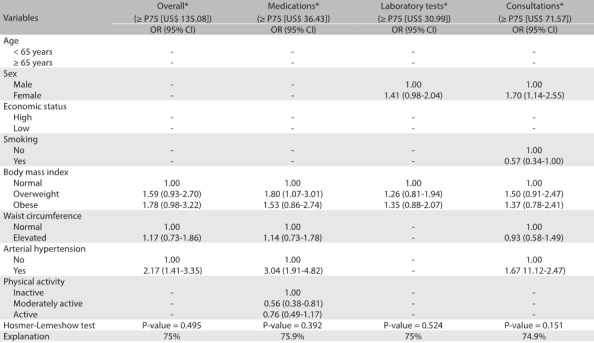
Adjusted model for association between high public healthcare expenditure and independent variables in Bauru (SP), Brazil (n = 963)

OR = odds ratio; 95% CI = 95% confidence interval; *model simultaneously adjusted for independent variables with P-value < 0.05 from chi-square test plus number of consultations, number of medicines and diagnoses of diabetes and dyslipidemia.

**Figure 1:**
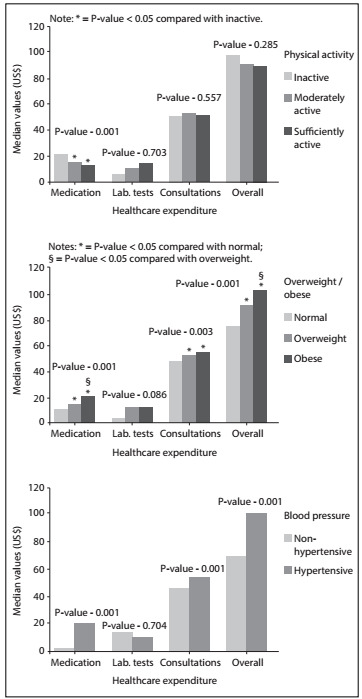
Healthcare expenditure according to physical activity, obesity and blood pressure among adults in Bauru (SP), Brazil (2010).

Figure 1 presents healthcare expenditure according to lifestyle behavior. Participants who were insufficiently active (Figure 1, Panel A), obese (Figure 1, Panel B) and hypertensive (Figure 1, Panel C) had the highest proportions of expenditure over a 12-month period.

## DISCUSSION

This study involved outpatients attended through the Brazilian National Health System in a medium-sized Brazilian city and investigated the correlates of public expenditure on healthcare services, specifically regarding consultations, laboratory tests and medications. Higher healthcare expenditure was associated with hypertension, overweight, sex and physical inactivity.

The presence of hypertension was associated with higher healthcare expenditure in three different categories (total expenditure, medication dispensed and consultations), thus denoting that this disease gave rise to a significant burden in relation to economic outcomes. This finding corroborates previous evidence that showed that cardiovascular diseases present high economic burdens in developing nations^1^. This result can at least partly be explained by the fact that management of hypertension is expensive, involving regular consultations and laboratory tests, as well as use of antihypertensive drugs.^20^

Additionally, overweight showed an association with increased healthcare expenditure relating to medication dispensed. Higher expenditure on medication among obese subjects might result from the link between obesity and a wide variety of cardiovascular and metabolic diseases.^21^ For the period 2008-2010, it has been estimated that the Brazilian Government’s expenditure on diseases relating to overweight/obesity was US$ 2.1 billion per year, of which US$ 1.4 billion was due to hospitalizations.^2^ In fact, overweight and obesity accounted for 6.8% and 9.3% of all hospitalizations among Brazilian men and women, respectively.^22^ Brazilian expenditure on bariatric surgery also increased from US$ 9.4 million in 2008 to US$ 17.4 million in 2011.^23^ A similar pattern has been observed in developed nations.^24^^,^^25^ Our findings also point out that primary care services have strategic relevance regarding preventive actions aimed towards controlling obesity and its future complications.

Physical inactivity is a major public health problem of the 21^st^ century.^26^ It is noteworthy that physical activity has a role in reducing the prevalence of obesity and related diseases, such as hypertension.^27^^,^^28^^,^^29^ Physical inactivity and abdominal obesity can act synergistically towards increasing public healthcare expenditure.^6^ Although physical inactivity was found in our study to be associated only with healthcare expenditure relating to medication dispensed, it should be emphasized that 1-4% of the total direct cost of healthcare is attributable to diseases relating to physical inactivity.^6^^,^^30^ Moreover, considering the role of regular physical activity on blood pressure and weight control, implementation of programs that promote physical activity in the context of the Brazilian National Health System can contribute significantly towards reducing costs.^5^^,^^31^^,^^32^

The higher expenditure relating to medical consultation that was observed among women is a pattern that has been found worldwide. Especially after the menopause, women use healthcare services more than men do.^13^^,^^33^^,^^34^ Regarding the healthcare services used by women, preventive care and therapeutic treatment are the ones most commonly used,^34^ and these services were available in the facilities analyzed in the present survey.

A number of limitations of this study need to be recognized. The cross-sectional design does not support statements of causality between health expenditure and its correlates. Moreover, only direct health care costs were taken into account in this study, while other sources of indirect costs were not considered (e.g. maintenance of the facilities and the size of healthcare professionals’ paychecks). It is important to recognize that we only included the biggest healthcare units in a single medium-sized city in Brazil. Thus, our results may not reflect other scenarios and should be extended to other settings only with caution. Other limitations to be considered are that the medical records were usually of poor quality; there was no information about medications obtained from other healthcare units or bought through government subsidy; and no measurements were made regarding adherence to treatments. Although the medical records constitute an important source of information regarding direct costs within primary healthcare, the bias caused through handwritten prescriptions issued by healthcare professionals and whether patients are using their medications correctly need to be considered. Additionally, selection of individuals who had had medical consultations during the previous six months may have biased the results from this study, since not all contacts with the healthcare unit are for medical consultations, and some patients may have less frequent visits. Physical activity was assessed through questionnaires, while other methods might have better accuracy (e.g. accelerometers and pedometers). Lastly, we did not have any information about alcohol consumption, which might have been an important risk factor for consideration as a potential confounder.

## CONCLUSIONS

Our findings showed that overweight, physical inactivity and arterial hypertension constituted independent risk factors associated with higher healthcare expenditure in relation to medication use, while arterial hypertension affected not only the overall expenditure, but also the expenditure attributed to medical consultations. Finally, sex seemed to be an important correlate of healthcare expenditure relating to medical consultations.
